# Supplementary material for: Complex‐centric proteome profiling by SEC‐SWATH‐MS
Source: Mol Syst Biol. 2019 Jan 14;15(1):e8438. doi: 10.15252/msb.20188438 (PMC6346213; doi:10.15252/msb.20188438)
Supplement: Supplementary file 8 — Dataset EV7 [file MSB-15-e8438-s008.zip › feature_plots_string/Q14203.pdf]

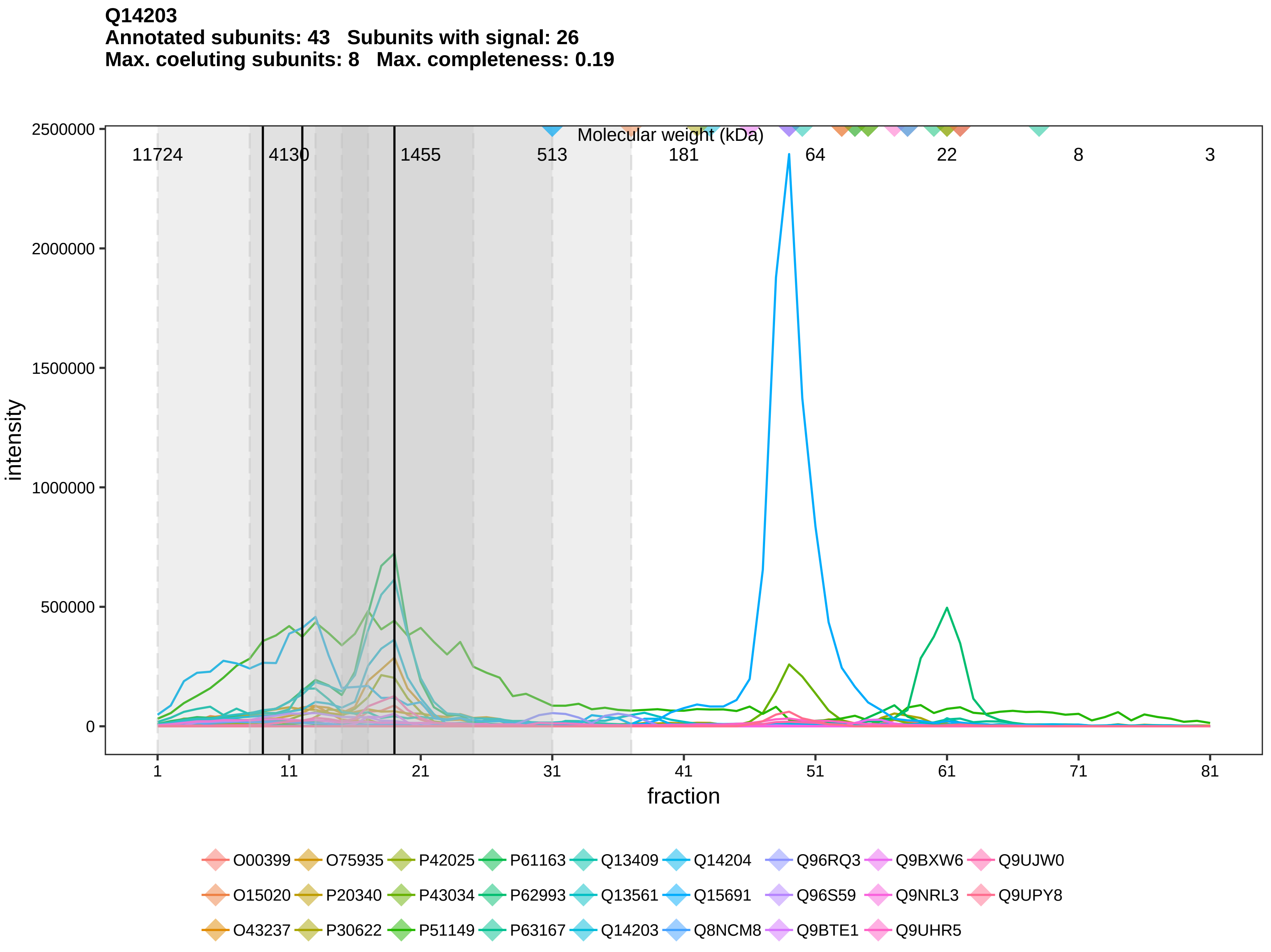

Q14203
Annotated subunits: 43 Subunits with signal: 26
Max. coeluting subunits: 8 Max. completeness: 0.19
Molecular weight (kDa)
11724
4130
1455
513
181
64
22
8
3
intensity
2500000
2000000
1500000
1000000
500000
0
fraction
1
11
21
31
41
51
61
71
81
O00399
O75935
P42025
P61163
Q13409
Q14204
Q96RQ3
Q9BXW6
Q9UJW0
O15020
P20340
P43034
P62993
Q13561
Q15691
Q96S59
Q9NRL3
Q9UPY8
O43237
P30622
P51149
P63167
Q14203
Q8NCM8
Q9BTE1
Q9UHR5
